# An update on freshwater gastropods serving as intermediate hosts of amphistomes infecting livestock in South Africa

**DOI:** 10.3389/fvets.2026.1862617

**Published:** 2026-10-12

**Authors:** Ignore Nyagura, Mokgadi Pulane Malatji, Sophy Nukeri, Madeline Siyasiziwe Sibula, Philile Ignecious Ngcamphalala, Samson Mukaratirwa

**Affiliations:** 1 School of Agriculture and Science, College of Agriculture, Engineering and Science, University of KwaZulu-Natal, Durban, South Africa; 2 National University of Science and Technology, Bulawayo, Zimbabwe; 3 One Health Centre for Zoonoses and Tropical Veterinary Medicine, Ross University School of Veterinary Medicine, Basseterre, Saint Kitts and Nevis

**Keywords:** amphistomes, detection, distribution, freshwater snails, identification, infection, South Africa

## Abstract

Improved surveillance of amphistome parasites and their intermediate hosts is crucial for comprehending their epidemiology and developing effective prevention and control measures, since amphistomosis is among the neglected parasitic diseases of ruminants, alongside schistosomiasis and fasciolosis. This study characterized the diversity of freshwater gastropods collected from six South African provinces and assessed their potential in the transmission of amphistomes infecting livestock. A total of 472 freshwater gastropods were collected from 6 provinces of South Africa between 2021 and 2023, and 11 snail species were identified based on shell morphology and confirmed using the mitochondrial cytochrome c oxidase I (COI) gene. Snails were screened for amphistome infection by cercarial shedding, followed by polymerase chain reaction (PCR) using the internal transcribed spacer 2 (ITS-2) region. Of these, 43 snails comprising *Radix (R.) natalensis* (*n* = 15), *Physella (P.) acuta* (*n* = 20), and *Melanoides (M.) tuberculata* (*n* = 8) shed cercariae. DNA was extracted from the shed cercariae and successfully amplified for amphistome DNA. However, only seven sequences obtained from cercariae shed by *P. acuta* (*n* = 5) and *M. tuberculata* (*n* = 2) were identified as amphistome spp. *Cercariae from P. acuta* were identified as *Calicophoron microbothrium* (*n* = 2) and Paramphistomidea spp. (*n* = 3), while the two cercarial samples from *M. tuberculata* were also identified as *C. microbothrium.* Whole tissue from the remaining 429 snails that did not shed cercariae was harvested and screened for amphistome DNA. Of these, 59 snails yielded an amplicon of the expected band size (380 bp) for amphistomes; however, the majority failed downstream quality control, while some amplified gastropod DNA. To the best of our knowledge, these findings provide the first confirmation of *M. tuberculata* as an intermediate host in the natural transmission of *C. microbothrium* in South Africa, as well as the first report of natural infection with *C. microbothrium* in *P. acuta* in sub-Saharan Africa. This study therefore represents a significant addition of this invasive snail species to the list of known intermediate hosts of amphistomes in Africa.

## 1 Introduction

Gastropoda is one of the most diverse and largest classes within the phylum Mollusca (1, 2). Traditional classifications divided gastropods into groups such as Prosobranchia, Pulmonata, and Opisthobranchia based on morphological characteristics and habitat preferences (3); however, these groupings are now considered outdated. Modern phylogenetic studies, particularly the clade-based system, have reclassified gastropods into six major clades: Patellogastropoda, Vetigastropoda, Cocculiniformia, Neritimorpha (Neritopsina), Caenogastropoda, and Heterobranchia (4). Of these, Caenogastropoda and Heterobranchia are the most ecologically diverse clades and include representatives in freshwater, marine, and terrestrial environments (4).

Of approximately 409 recognized gastropod families worldwide, 33 families, consisting of approximately 5,000 species, are found in freshwater habitats (5). These gastropods play a crucial role in the transmission dynamics of trematodes, acting as intermediate hosts (IHs) (6). Among the trematodes that use freshwater gastropods as IHs are amphistomes, which are known to cause amphistomosis, a neglected tropical disease of livestock (7). The range and diversity of these amphistome species vary widely across continents, and their impact is especially significant in Africa, where they are reported to cause greater economic losses in livestock (7). However, studies related to the epidemiology of the disease and species of gastropods acting as IHs are lagging compared to well-known parasites such as schistosomes and fasciolids (8).

Amphistomes primarily affect ruminants and use various freshwater gastropods as intermediate hosts in different continents (9–14). Freshwater gastropod species from the families Ampullariidae, Lymnaeidae, Paludomidae, Physidae, Planorbidae, Pomatiopsidae, Racidinae, and Thiaridae have been documented in various regions of South Africa (15–18), including the 10 confirmed gastropod species as intermediate hosts of known amphistomes in Africa, namely *Bulinus (B.) (Physopsis) globosus*, *B. (Physopsis) nasutus*, *B. tropicus*, *B. forskalii*, *B. senegalensis*, *B. mairei*, *B. liratus*, *Biomphalaria (Bio.) pfeifferi*, and *Anisus (Ceratophallus) natalensis,* and *Galba (Lymnaea) truncatula* (6). Recent studies have added *Physella (P.) acuta* (9), *Melanoides tuberculata* (19), and *Segmentorbis* (20) to the list of intermediate hosts of amphistomes in Africa. While these findings provide some insights into amphistome transmission, the broader distribution of amphistome infections across African freshwater gastropods remains poorly understood as the majority of the studies is conducted in the eastern region of Africa (6). Furthermore, despite the growing importance of amphistomosis, the understanding of the spatial distribution of these parasites and intermediate hosts in Africa remains limited, with few studies primarily confined to just 10 African countries to date (6, 19, 21). This is likely due to limited funding, lack of expert interest, and a research focus on other trematodes such as *Fasciola* and *Schistosoma*. Additionally, ecological factors such as invasive gastropod species, climatic variability, and the vast unsurveyed freshwater systems in many African countries further hinder the comprehensive understanding of the epidemiology of amphistomosis (7).

This is the case in South Africa, where information on amphistome infections in both intermediate host gastropods and vertebrate hosts is limited. Before the report of Paramphistomoidea spp. infection in *P. acuta* (21), amphistome infections in intermediate hosts were reported in the early 1900s and 2000s, with only *B. tropicus* reported as the natural intermediate host of *Calicophoron calicophorum* and *C. microbothrium* (22–24). Therefore, this study aims to address the information gap by using molecular techniques to detect amphistome infections in freshwater gastropods and further barcode the diversity of the gastropods in South Africa. This will complement the use of traditional morphological identification methods that are limited by the presence of cryptic species, morphological plasticity of the shell, and potential hybridization (25–28).

## 2 Materials and methods

### 2.1 Sample collection

A gastropod survey was conducted across six provinces of South Africa (Supplementary Table S1), and a total of 472 gastropods were collected from several habitats in KwaZulu-Natal (*n* = 60), Limpopo (*n* = 166), Free State (*n* = 45), Mpumalanga (*n* = 115), Gauteng (*n* = 26), and Eastern Cape (*n* = 60), using large scoops or hand-picking, following standard freshwater snail sampling procedures as described by Brown (29). Gastropods were morphologically identified to species level *in situ* following the classification keys described by Brown (29), then individually placed in small cups with distilled water and exposed to light followed by darkness for one hour each to stimulate cercarial shedding. The shedding of cercariae was performed as described by Chingwena et al. (30), and their presence was confirmed under a Zeiss Stemi 305 stereo microscope (Zeiss Microscopy, Germany). The presence or absence of cercariae was recorded, after which the shed cercariae specimens were immediately preserved for subsequent molecular characterization. A total of 43 gastropods from Limpopo (*R. natalensis, n* = 15), Gauteng (*P. acuta*, *n* = 20), and Mpumalanga (*M. tuberculata*, *n* = 8) released cercariae. Cercariae from each shedding gastropod were transferred into individual falcon tubes, and the gastropods were euthanized in 30% ethanol and preserved in 70% ethanol for future molecular analysis.

### 2.2 DNA extraction

Whole gastropod tissues were harvested from 429 non-shedding snails, individually rinsed with nuclease-free water, and patted dry with absorbent paper to remove excess ethanol for molecular analyses. DNA was extracted using the Genomic DNA™ Tissue MiniPrep kit (Zymo Research Corporation, California, USA) following the manufacturer’s instructions, with a modified overnight incubation. Cercariae were centrifuged at 1,500 × g for 10 min to concentrate the parasites. The supernatant was discarded, and the cercarial pellet was washed by resuspending it in 500 μL of sterile water, vortexing for 30 s, and centrifuging it twice for 5 min each time to remove any debris. The cercarial DNA was extracted using the Qiagen DNeasy Blood and Tissue Kit (Qiagen, Germany) following the manufacturer’s protocol. Genomic DNA was quantified using a NanoDrop spectrophotometer and stored at −20 °C.

### 2.3 Molecular identification of gastropods

The mitochondrial region of the gastropod DNA was amplified using the universal mitochondrial cytochrome c oxidase subunit I (COI) primers LCO1490: 5’-GGTCAACAAATCATAAAGATATTGG-3′ and HCO2198: 5’-TAAACTTCAGGGTGACCAAAAAATCA-3′ (30). Amplification was performed in a 25 μL polymerase chain reaction (PCR) mixture containing 12 μL of 2 × Taq DNA Polymerase Master Mix RED™, 1 μL of each primer (10 μM), approximately 20–50 μg/μL of template DNA, and nuclease-free water to a final volume of 25 μL. Thermal cycling conditions were set at 95 °C for 5 min, followed by 40 cycles of 60 s at 95 °C, 70 s at 50 °C, and 60 s at 72 °C a final extension at 72 °C for 10 min. Fragments were separated using 2% agarose gels stained with SYBR Safe DNA gel stain (Applied Biosystems, USA) and visualized using Uvidoc HD6 (Uvitec, Cambridge) with a band size of 710 bp.

Before cycle sequencing, the amplicons were cleaned up and purified using a combination of 2 μL of FastAP™ and 1 μL of Exonuclease enzymes (Applied Biosystems, USA), and the purification reaction was performed at 37 °C for 15 min, followed by 85 °C for 15 min. Cycle sequencing was performed in both forward and reverse directions in a final reaction volume of 10 μL containing 0.7 μL BigDye, 2.55 μL sequencing buffer, 0.75 μL ddH_2_O, 1 μL of primer, and 5 μL of the PCR product, following cycling conditions: one cycle at 95 °C for 2 min; 40 cycles at 85 °C for 10 s, 53 °C for 10 s, and 60 °C for 2 min 30 s. Sequencing was performed using the BigDye Terminator™ v3.1 cycle sequencing kit (Thermo Fisher Scientific, California, USA) based on the Sanger chain-termination method, and both forward and reverse sequences were visualized using the 3,130 Genetic Analyzer (Thermo Fisher Scientific, California, USA).

### 2.4 Molecular detection of amphistome infections in gastropods and identification of cercariae

The nuclear ribosomal DNA (rDNA) internal transcribed spacer 2 (ITS-2) region was amplified using the primers GA1 (5’-AGAACATCGACATCTTGAAC-3′) and BD2 (5’-TATGCTTAAATTCAGCGGGT-3′) (20) to detect amphistome DNA in gastropods and cercariae. Amplification was performed in a 25 μL reaction mixture consisting of 12.5 μL of One Taq Quick-Load 2 × Master Mix (New England Biolabs), 1 μL of each primer (10 μM), 7.5 μL of nuclease-free water, and 3 μL (approximately 15–40 μg/μL) of the template DNA. PCR reactions included *C. microbothrium* DNA as a positive control and nuclease-free water as a negative control. The cycling conditions were set with an initial denaturation at 95 °C for 3 min, followed by 30 cycles of denaturation at 95 °C for 30 s, annealing at 52 °C for 45 s, and extension at 72 °C for 1 min, with a final extension step at 72 °C for 15 min. The amplicons were electrophoresed on a 2% agarose gel, stained with SYBR Safe DNA Gel Stain (Applied Biosystems, USA) and visualized using Uvidoc HD6 (Uvitec, Cambridge), and successful amplification was indicated by a band at 380 bp. The amplicons were then sent to Inqaba Biotechnical Industries (Pretoria, South Africa) for bidirectional Sanger sequencing.

### 2.5 Phylogenetic relationships of gastropods and amphistomes detected from gastropod samples

The forward and reverse sequences were visualized, assembled, and manually edited to address ambiguous positions and generate a consensus sequence using BioEdit version 7.2 (Sequence Alignment Editor) (31). Clustal W was used to align the sequences with the closest homologous sequences obtained from the GenBank database. The sequences were then trimmed at both ends to a consistent length of 489 nucleotides for snails and 365 nucleotides for amphistomes. Tamura 3-parameter (T92) and Hasegawa-Kashino-Yano (HKY) models were selected as the best-fit models for the snail and parasite datasets, respectively. The neighbor-joining (NJ) and maximum-likelihood (ML) trees were generated using MEGA 7 software (32). Phylograms were generated using a 50% majority-rule consensus, and nodal support was evaluated through 1,000 bootstrap pseudo-replicates in the order ML/NJ.

## 3 Results

### 3.1 Identification and distribution of the collected gastropods

The COI marker confirmed the morphological identification of *Pseudosuccinea (Ps.) columella* and *R. natalensis* from the Lymnaeidae family, *M. tuberculata* and *Tarebia (T.) granifera* from the Thiaridae family, *Physella* (*P.*) *acuta* from the Physidae family, *Bulinus (B.) truncatus*, *B. tropicus*, *B. forskalii, Biomphalaria (Bio.) pfeifferi*, and *Gyraulus (G.) connollyi* from the Planorbidae family (Figure 1). Despite several attempts, sequencing of *B. globosus* based on COI failed several times; however, this species was genetically confirmed using the 16S rRNA gene (results not presented). The maximum-likelihood tree revealed distinct cladal clustering representing different families, although weakly supported except for the Thiaridae family which showed a strongly supported clade (ML/NJ: 99/100). The Thiaridae clade represented *M. tuberculata* from Limpopo province and *T. granifera* from both Mpumalanga and KwaZulu-Natal provinces of South Africa. The weakly supported Planorbidae clade formed several subclades that clearly distinguished between different genera and species. The *Bulinus* genus formed three separate well-supported sister clades, showing the close genetic relationship between *B. tropicus* and *B. truncatus* complex, which were also closely related to *B. forskalii* as compared to the GenBank-derived *B. globosus*. The Lymnaeidae clade also showed a clear distinction between *Ps. columella* and *R. natalensis*, which were the only lymnaeid species identified. The sequences were submitted to GenBank under the accession numbers PV065487-PV065516 and PQ898671-PQ898695.

**Figure 1:**
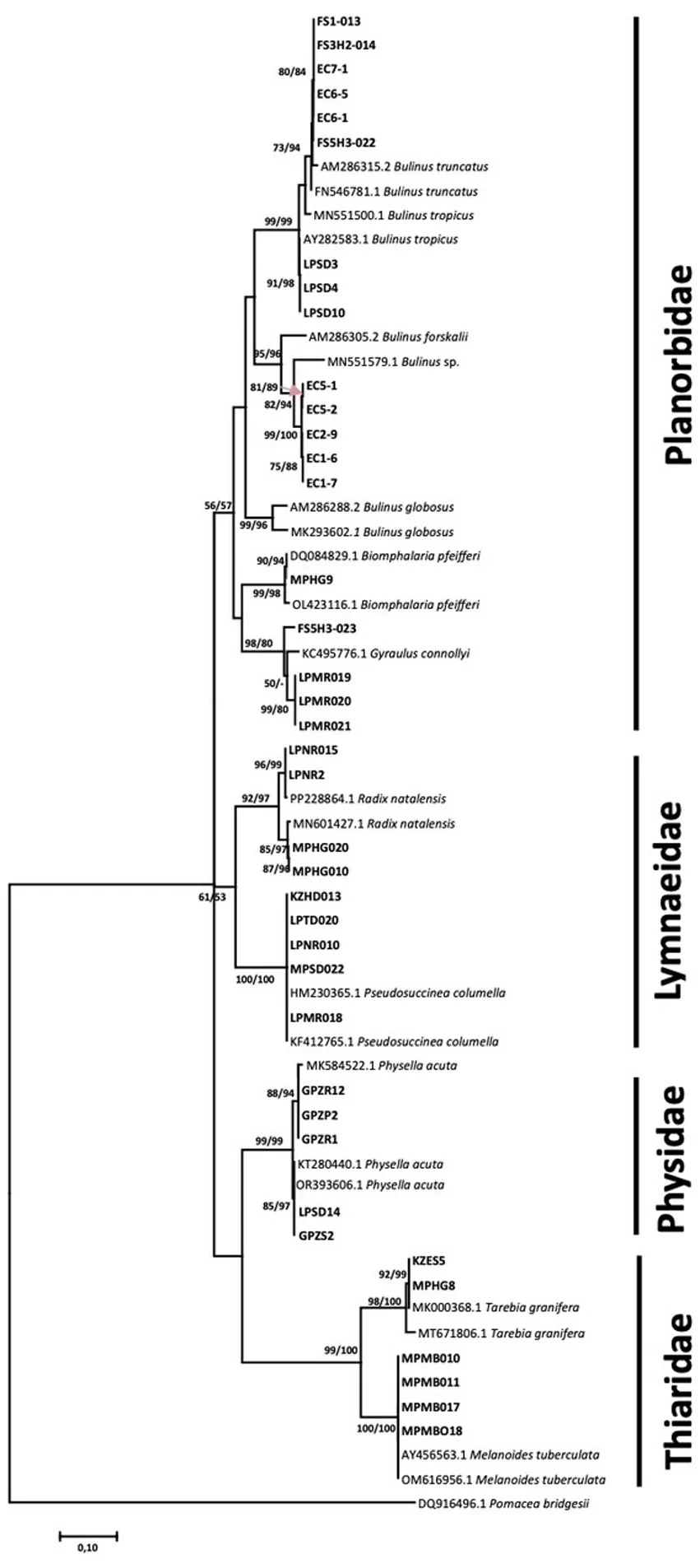
Maximum likelihood tree illustrating the relationship freshwater snails different provinces from South Africa based on the CO1 gene. The bootstrap values are shown on the node in the order ML/NJ, and study samples are presented in bold; LP- Limpopo province, MP, Mpumalanga province; GP, Gauteng province; FS, Free State; EC, Eastern Cape; KZ, KwaZulu-Natal.

### 3.2 Molecular detection and characterization of amphistomes

Out of 472 gastropods that were examined, 73 (15.47%) yielded an amplicon of the expected size (approximately 380 bp), which was in line with the size of the amphistome DNA used as a positive reference (59/429 for dissection and 14/43 for cercariae). Amphistome infection, however, was only considered confirmed following sequence-based species verification. Cercariae were detected from *P. acuta*, *M. tuberculata* and *R. natalensis*. No bands were detected in *T. granifera*, *Ps. columella, B. globosus*, *B. forskalii,* and *G. connollyi* in this study. However, sequencing was successful only for 10 samples from *P. acuta* and *M. tuberculata* cercariae, and *B. truncatus* tissue, while others produced poor-quality or mixed sequencing chromatograms and non-specific amplification products. In addition, some sequences matched gastropod DNA rather than amphistome DNA, preventing reliable species identification.

Sequences from *P. acuta* (sample ID: PH69A, 5G) and *M. tuberculata* (ME48, 9B) cercariae were identified as *C. microbothrium*. The isolates showed 100% homology with *C. microbothrium* sequences from Zimbabwe (KP639632) and Egypt (OK216192), which formed a strongly supported clade (98/96) but showed weak support with other *Calicophoron* species (Figure 2). Sequences from *P. acuta* (sample ID: 1G, 4G, 6G) also showed a 100% similarity to Paramphistomoidea sp. from Kenya (KX668932). Although the Paramphistomoidea spp. clade was strongly supported, the resolution within the clade containing the studied isolates was moderately supported. Furthermore, the distant relationship between these isolates and *Calicophoron, Carmyerius, Gastrothylax*, and *Paramphistomum* species highlights that although these isolates from Gauteng province were amphistomes, they may belong to a different genus and possibly a different family from those represented by the above-mentioned taxa. These sequences were submitted to GenBank under the accession numbers PV073218-PV0732224.

**Figure 2:**
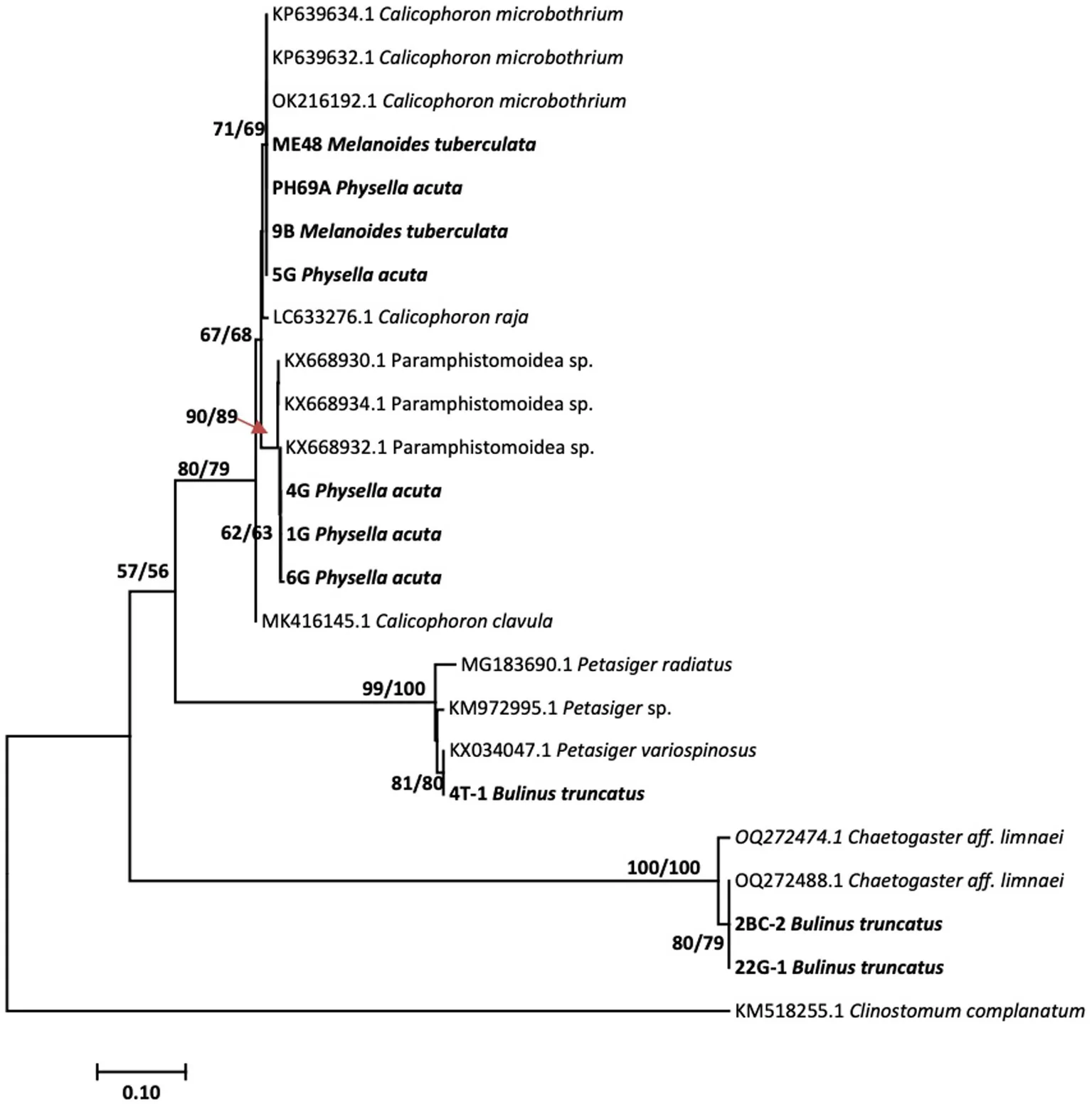
Maximum likelihood tree depicting the relationship between amphistomes and other trematode species detected in freshwater snails provinces from South Africa based on the ITS-2 gene. The bootstrap values are shown on the node in the order ML/NJ, and study samples are presented in bold.

*Bulinus truncatus* had trematode DNA from the family Echinostomatidae, identified as *Petasiger variospinosus* (4 T-1, accession number: PV073225), and DNA from another snail parasite, *Chaetogaster aff. limnaei* (accession number: PV073226) (Figure 2). These formed strongly supported clades with other GenBank-derived sequences.

## 4 Discussion

To date, 13 freshwater snail species have been confirmed to act as intermediate hosts of 10 amphistome species across seven African countries (6, 19, 33). In addition, unidentified Paramphistomoidea spp. have also been detected in *P. acuta* (21) and *Bio. pfeifferi* (20) in South Africa and Kenya, respectively. Eleven freshwater gastropods were screened for amphistome infections in this study based on the ITS-2 region, which was selected due to its extensive utilization for molecular identification and phylogenetic studies of trematodes owing to its high interspecific variability and the availability of substantial reference sequences in GenBank (20, 34–36). Compared with more conserved markers such as 18S and 28S rDNA, the ITS-2 generally provides greater resolution for discriminating closely related amphistome species. However, the present study demonstrated that its application to whole snail tissues has limitations.

Although 59 snail tissue and 14 cercariae samples yielded amplicons of the expected ITS-2 size, only 10 high-quality sequences suitable for species identification were generated. Consequently, amphistome infection status was based solely on sequence-confirmed identifications. Several PCR products were identified as gastropod ITS sequences, indicating non-specific amplification or co-amplification of host DNA when the ITS-2 assay was applied directly to whole snail tissues. This methodological limitation reduced the specificity of the assay and contributed to the low sequencing success observed. Therefore, the molecular detection rate based on PCR-positive snail tissue samples should be interpreted with caution, and PCR amplicons of the expected size should not be regarded as definitive evidence of amphistome infection without sequence confirmation. Similar challenges were reported in studies (21, 37), in which the ITS-2 primers often lacked sufficient discrimination power in invertebrate studies. Despite these limitations, the primers have been validated as effective molecular markers for amplifying direct parasite DNA from adult amphistome tissues and egg stages in various studies (20, 34–36). A similar methodological limitation was also encountered during the molecular identification of freshwater gastropods. Although the COI marker successfully confirmed most gastropod species, repeated amplification failures were observed for *B. globosus*, requiring confirmation using the mitochondrial 16S rRNA marker. This finding indicates that no single molecular marker is universally applicable across all freshwater gastropod taxa and highlights the importance of using complementary genetic markers when amplification with the primary barcode is unsuccessful.

The 11 snail species screened included seven species that have previously been demonstrated to act as IHs of amphistomes in livestock (33), except for *R. natalensis*, *G. connollyi*, *T. granifera* and *B. truncatus*. Surprisingly, none of the Planorbidae gastropods, which are known as the main IHs of amphistomes in some African countries (6, 20, 33), including *B. globosus*, which showed a high infection rate in Zimbabwe, and *B. tropicus*, which has been previously implicated in the transmission of *Calicophoron calicophorum* and *C. microbothrium* in South Africa (22, 23), had amphistome DNA or shed amphistome cercariae. Despite several freshwater gastropod species showing amplification for trematode infections, amphistome infections in this study were only confirmed in *M. tuberculata* and *P. acuta* from Mpumalanga and Gauteng provinces, respectively. Although this is the first report of natural infection of *C. microbothrium* in these two gastropod species in South Africa, natural infections of *M. tuberculata* with this *Calicophoron* species were recently reported in Zimbabwe (19). Furthermore, earlier experimental susceptibility studies demonstrated this in Zimbabwe (30) and South Africa (38). Moreover, *M. tuberculata* has been reported to be susceptible to *Gastrodiscus aegyptiacus*, a fluke of equids, pigs, and warthogs reported in South Africa (39). This study confirmed and reiterated the ability of *M. tuberculata* to act as a natural IH and to naturally transmit *C. microbothrium*, as it was detected in shed cercariae.

*Physella acuta* was previously considered a freshwater species of no veterinary or medical importance (40). However, recent evidence of natural infections of this invasive species with *F. hepatica* in southern Argentina (41) and France (27) has shown the importance of this invasive gastropod in the transmission of trematodes. Furthermore, recent studies in South Africa also detected *F. hepatica* DNA in *P. acuta* (21), although this finding does not confirm that the gastropod can naturally transmit the parasite. In our study, PCR and sequencing of cercariae shed by *P. acuta* confirmed natural infection of the gastropod with *C. microbothrium* and Paramphistomidae spp. in southern Africa for the first time. Paramphistomidae spp. infections further supplement recent reports of this amphistome DNA in *P. acuta* in Gauteng province, South Africa (21).

This unidentified *Paramphistomidae* sp. was closely related to an isolate previously detected in *Biomphalaria pfeifferi* in Kenya (20), while showing more distant relationships to species within the genera *Calicophoron*, *Carmyerius*, *Gastrothylax*, and *Paramphistomum* (42). Notably, high sequence similarity was also observed between this *Paramphistomoidea* sp. and isolates morphologically identified as *Leiperocotyle gretillati* from wild ruminants in Zimbabwe, which constituted the first report of this species in southern Africa, following its original description based on specimens from the Democratic Republic of Congo (43). Collectively, these findings highlight the capacity of *P. acuta* to support the development of amphistome larval stages (21) and underscore its potential role in the transmission of multiple amphistome species in South Africa.

Nonetheless, *B. tropicus*, which has been previously implicated in the transmission of *C. calicophorum* and *C. microbothrium* in South Africa (22, 23), Zambia (44), and Kenya (45), showed no amphistome infection in this study. Similar results were obtained with *Bio. pfeifferi*, which has been implicated in the transmission of *Calicophoron sukari* in Ethiopia (46) and Kenya (44), and Paramphistomidea spp. in Kenya (20). While previous reports have shown that *R. natalensis* is refractory to *C. microbothrium* (30), this lymnaeid snail species was found to be infected with *Gastrothylax*/*Paramphistomum* in Zimbabwe (47). However, our study was consistent with (19), in which amphistome DNA was also not detected in *R. natalensis*.

Another trematode species was detected in *B. truncatus* and identified as *Petasiger variospinosus. Petasiger* species are cosmopolitan parasites that use freshwater snails as first intermediate hosts, while piscivorous birds and mammals are the main definitive hosts (48, 49). Previous studies have reported *Petasiger* species in *Burnupia transvaalensis* (50), *Burnupia trapezoides* (51), and *Burnupia mooiensis* (52), all of which were collected from the Vaal River and Limpopo River systems in South Africa (53). This suggests that *Petasiger* sp. may have a broader geographic distribution than previously recognized, potentially affecting a wider range of freshwater snail species across different aquatic systems.

Molecular analysis also detected the DNA of *Chaetogaster aff. Limnaei*, a snail parasite belonging to the segmented worms (Annelida), in *B. truncatus*, representing the first recorded occurrence of this species in South Africa. As a cosmopolitan predator, *Ch. limnaei* has been documented to parasitize a diverse range of freshwater snails, including those within the families Lymnaeidae, Physidae, and Planorbidae (54, 55), all of which were observed in this study. Thus, the detection of *Petasiger* sp. and *Chaetogaster aff. Limnaei* in this study, together with that of *Paragonimus* spp. in (19), supports the suggestion that various gastropod species in African freshwater systems can serve as hosts for a diverse range of trematodes and other parasites and thus calls for further investigation of the full range of trematode species transmitted by the South African snail species.

## 5 Conclusion

This study highlights the ecological diversity of freshwater gastropod snails in South Africa and their potential role in the transmission of amphistome parasites. Notably, it provides the first report of natural infection of *M. tuberculata* and *P. acuta* with *C. microbothrium* in South Africa. The detection of *C. microbothrium* and *Paramphistomoidea* spp. in *P. acuta* further identifies this species as a potentially important intermediate host of amphistomes in the region. Given that *P. acuta* is an invasive and widely distributed species in South Africa, its involvement in amphistome transmission presents additional challenges for the development of effective strategies to manage and control parasitic infections in snail populations, particularly in areas where amphistomes pose a significant risk to livestock. Consequently, future research should focus on defining the full geographic distribution of these freshwater gastropod species and identifying additional areas where they may act as intermediate hosts for amphistomes. Such efforts should include systematic screening for infection through cercarial shedding, complemented by molecular tools to accurately identify amphistome species involved in transmission.

## Data Availability

All relevant data generated or analysed during the study will be provided when requested. The sequences were submitted on GenBank under the accession numbers PV065487-PV065516, PQ898671-PQ898695, PV073218 PV0732224, PV073225-PV073226.
