# Supplementary material for: An update on freshwater gastropods serving as intermediate hosts of amphistomes infecting livestock in South Africa
Source: Front Vet Sci. 2026 Oct 12;13:1862617. doi: 10.3389/fvets.2026.1862617 (PMC13696199; doi:10.3389/fvets.2026.1862617)
Supplement: Supplementary file 1 [file Table_1.docx]

**Supplementary Table S1.** Details of freshwater snail collection sites, habitat types, snail species collected, and GPS coordinates in South Africa.

| Province | City | Habitat type | Snail species collected | GPS coordinates |
| --- | --- | --- | --- | --- |
| KwaZulu-Natal | Durban | Dam | *Ps. columella, P. acuta* | -29 5769826, 31 1001453 |
| KwaZulu-Natal | Eshowe | Pond | *T. granifera* | -28 830134, 31 496850 |
| KwaZulu-Natal | Mhlathuze | River | *T. granifera* | -28 73697, 31 50657 |
| Free State | Harrismith | Pond | *G. connollyi, B. truncatus* | -27 202500, 26 9100833 |
| Free State | Harrismith | Pond | *B. truncatus* | -28 2348889, 29 0879167 |
| Free State | Harrismith | Pond | *G. connollyi, B. truncatus* | -28 2496389, 29 0804444 |
| Free State | Harrismith | Pond | *B. truncatus* | -27 9443611, 27 5652222 |
| Mpumalanga | Mbombela | Dam | *R. natalensis, P. acuta, M. tuberculata, B. globosus, Bio. Pfeiferri* | -25 741012, 28 209156 |
| Mpumalanga | Mbombela | Dam | *M. tuberculata* | -25 403317, 30 98328 |
| Mpumalanga | Middleburg | Pond | *Ps. columella* | -25 562861, 29 279495 |
| Mpumalanga | Middleburg | Pond | *P. acuta* | -25 562861, 29 279496 |
| Limpopo | Tzaneen | Dam | *Ps. columella, R. natalensis, B. tropicus, B. globosus* | -24 104562, 30 244393 |
| Limpopo | Tzaneen | River | *T. granifera, B. globosus* | -24 040476, 30 312355 |
| Limpopo | Tzaneen | River | *Ps. columella, R. natalensis, G. connollyi, B. globosus, Bio. Pfeiferri* | -24 040476, 30 312355 |
| Limpopo | Polokwane | Stream | *P. acuta, B. tropicus* | -23 846993, 29 710013 |
| Eastern Cape | Mthata | Pond | *Ps. columella* | -31 4829722, 28 7644722 |
| Eastern Cape | Mthata | Pond | *B. forskali* | -31 5501667, 28 7316389 |
| Eastern Cape | Mthata | Pond | *B. truncatus, B. forskali* | -31 2943056, 28 7005833 |
| Eastern Cape | Mthata | River | *Ps. columella, B. forskali* | -31 6319167, 28 7552500 |
| Eastern Cape | Matatiele | Pond | *B. truncatus* | -31 4572500, 28 7545556 |
| Eastern Cape | Matatiele | Pond | *B. truncatus* | -30 6186944, 28 5460278 |
| Gauteng | Culinan | Stream | Ps. columella | -25 701277, 28 548923 |
| Gauteng | Culinan | Stream | *P. acuta* | -25 690314, 28 568356 |
| Gauteng | Culinan | Pan | *P. acuta* | -25 705189, 28 563196 |
| Gauteng | Pretoria West | River | *P. acuta* | -25 5279270, 28 2302769 |
